# Anticoagulation strategies in patients with coexisting traumatic intracranial hematomas and cerebral venous sinus thrombosis: an observational cohort study

**DOI:** 10.1007/s00701-024-06287-5

**Published:** 2024-09-27

**Authors:** Julia Antonsson, Charles Tatter, Anna Ågren, Peter Alpkvist, Eric Peter Thelin, Alexander Fletcher-Sandersjöö

**Affiliations:** 1 Department of Clinical Neuroscience, Karolinska Institutet, Bioclinicum J5:20, 171 64-Solna Stockholm, Sweden; 2 Department of Radiology, Stockholm Southern Hospital, Stockholm, Sweden; 3 Coagulation Unit, Hematology Centre, Karolinska University Hospital, Stockholm, Sweden; 4 Department of Neurosurgery, Karolinska University Hospital, Stockholm, Sweden; 5 Department of Neurology, Karolinska University Hospital, Stockholm, Sweden

**Keywords:** Traumatic brain injury, Dural sinus thrombosis, Cerebral venous sinus thrombosis, Intracranial hematoma, Anticoagulation

## Abstract

**Purpose:**

Post-traumatic cerebral venous sinus thrombosis (ptCVT) is a rare but serious complication of traumatic brain injury (TBI). Managing ptCVT is challenging due to the concurrent risk of traumatic intracranial hematoma (ICH) expansion. Limited data exists on the safety and efficacy of anticoagulation therapy (ACT) in these cases.

**Methods:**

This single-center observational cohort study included adult TBI patients with concurrent ICH and ptCVT. Low-molecular-weight heparin (LMWH) or heparin infusion was used to treat all ptCVTs based on institutional protocols. The outcomes of interest were hemorrhagic and thrombotic complications.

**Results:**

Out of 1,039 TBI-patients admitted between 2006 and 2020, 32 met the inclusion criteria. The median time from injury to ptCVT diagnosis was 24 h. ACT was initiated at a median of 9 h after ptCVT diagnosis. Patients were administered either heparin infusion (*n* = 8) or LMWH at dosages ranging from 28 to 72% of the therapeutic level (*n* = 24). There were no hemorrhagic complications, even in patients receiving LMWH at ≥ 50% of the therapeutic dose. Thrombotic complications occurred in 3 patients (9.4%) – two cases of thrombus progression and one venous infarct. The patients who developed thrombotic complications differed from those who did not by having a 17-h delay in ACT initiation after diagnosis or by receiving an initial LMWH dose at 28% of the therapeutic level.

**Conclusion:**

LMWH at approximately 50% of the therapeutic level was effective for managing ptCVT associated with TBI in our retrospective dataset, with no risk of hematoma expansion. Prospective trials are warranted to confirm these results.

## Introduction

Our previous research indicates that around 3% of patients with moderate-to-severe traumatic brain injury (TBI) will develop post-traumatic cerebral venous sinus thrombosis (ptCVT) during their index hospitalization [8]. The underlying mechanism includes direct trauma to or compression of the venous sinuses, often due to skull fractures and extra-axial hematomas [2, 14], as well as disturbances in hemostatic balance [14, 19]. For these patients, effective management is important to prevent venous hypertension, which can lead to raised intracerebral pressure, cerebral edema, capillary rupture with secondary hemorrhages, and venous infarction [2, 7, 12, 18].

In non-traumatic CVT, anticoagulation therapy (ACT) is the established treatment, typically involving low-molecular-weight heparin (LMWH) or direct-oral anticoagulants [7, 18]. However, in the case of ptCVT, which often develops in the first days following trauma, there is the added concern of potential traumatic intracranial hematoma (ICH) expansion [4, 8, 9]. This situation presents a clinical dilemma: how to prevent clot growth without increasing the risk of ICH progression?

The available data on the management of ptCVT in adults with coexisting ICH are limited, consisting of one cohort study [4] and five case reports [1, 6, 12, 14, 16]. The largest study involved 23 patients, none of whom received ACT, and found thrombus progression in 24% of those who underwent follow-up CT venography [4]. Among the case reports, one described a thrombosis progression with fatal outcome in a patient who did not receive ACT [6], another documented successful recanalization through conservative treatment in one patient [14], while the remaining reported on a total of six patients treated with heparin infusion, LMWH, or warfarin without any hematoma expansion [1, 12, 16] (Table 1). Other studies addressing ptCVT management have not provided detailed data on the impact that ACT had on hematoma expansion [11, 13, 15, 17, 19]. Overall, the existing evidence on treatment of ptCVT in conjunction with ICH is limited, with significant gaps in our understanding of the efficacy and safety of early ACT.

**Table 1:** Prior studies on treatment and outcomes for patients with coexisting ptCVT and ICH

Reference	Patients (n)	Received ACT (n)	Hematoma expansion after ACT (n)	ptCVT-related complication
Almandoz, 2010 [4]	23	0	N/A	thrombosis progression (*n* = 4)
Dobbs, 2012 [6]	1	0	N/A	death due to thrombosis (*n* = 1)
Grangeon, 2017 [12]	2	2 (heparin infusion)	0	-
Pescatori, 2017 [16]	1	1 (LMWH)	0	-
Afshari, 2018 [1]	4	3 (heparin infusion, LMWH, warfarin)	0	death due to thrombosis despite heparin infusion (*n* = 1)
Isan, 2022 [14]	1	0	N/A	-

Abbreviations: *ACT* anticoagulation therapy, *ICH* intracranial hematoma, *ptCVT* post traumatic cerebral venous thrombosis

Given this context, the aim of this study was to assess a consecutive cohort of patients with diagnosed ptCVT and ICH who were treated at an institution where ACT is the normative protocol.

## Methods

### Study design, study population and data collection

This was a single-center observational cohort study enrolling adults (≥ 15 years) with mild-to-severe TBI (Glasgow Coma Scale (GCS) 3–15) treated at the Karolinska University Hospital in Stockholm, Sweden between 2006 and 2020. We excluded those without a ptCVT, as well as those with incomplete data (primarily due to initial treatment at other centers) and known CVT prior to the trauma. The study hospital is the only level 1 trauma center equivalent in the region and offers neurosurgical and neuro-intensive care to a population of approximately 2.3 million with a geographically defined catchment area. Patients eligible for inclusion were identified using the Karolinska Neurotrauma Database, which includes all TBI-patients admitted to the hospital. Clinical data were extracted from electronic medical records using TakeCare (CompuGroup Medical Sweden AB, Farsta, Sweden) and Centricity Critical Care (GE Healthcare, Chicago, IL). Radiological data were reviewed using Sectra Picture Archiving and Communication System (PACS, IDS7, Sectra AB, Linköping, Sweden). Hematoma volumes were calculated using semi-automated threshold-guided planimetry, as previously described [9, 10]. The study was approved by the Swedish Ethical Review Authority (Dnr 2019–04446, amendment 2022–06135-02).

### Use of thromboprophylaxis and diagnosis of ptCVT

Pharmacological and mechanical thromboprophylaxis, including anti-embolism compression stockings or pneumatic compression devices, were used for all patients barring any contraindications. The timing of prophylactic LMWH initiation was determined by the neurosurgical and neurocritical care team, with a goal of initiating it within 48 h of trauma. Standard dosages for patients without ptCVT were 5,000 units of subcutaneous Dalteparin, 40 mg of Enoxaparin, or 4500 units of Tinzaparin, once daily.

Investigative procedures for diagnosing or ruling out a ptCVT were determined by the treating team. According to our standard management protocol, initial non-contrast computed tomography (CT) head scans were performed on admission, with follow-up scans typically obtained 6 h later and then according to the clinician’s judgment. CT venography or magnetic resonance imaging (MRI) venography was performed if there was a fracture or hematoma overlying a dural venous sinus, or any other clinical or radiological suspicion of a CVT. In cases where a ptCVT was diagnosed, the approach involved increasing the LMWH dosage, maintaining the current regimen, or transitioning to a heparin infusion. Treatment decisions were made collaboratively during patient rounds (or off-hours consultations), involving 1–2 neurosurgeons (always including at least one consultant), the ICU doctor responsible for the patient, and a senior ICU overseeing all patients in the unit, with input from a coagulation medicine specialist as needed. Regular monitoring with CT venography was usually maintained throughout the patient's hospitalization. Total duration of anticoagulation treatment varied between 3–6 months after initiation.

### Statistics

Shapiro Wilks test was performed to determine data distribution for continuous variables. As all continuous data showed a non-normally distributed pattern (Shapiro Wilks test p-value < 0.05), they are presented as median and interquartile range (IQR). Categorical data are presented as numbers (proportion). Data were stratified by the type of ACT, i.e., heparin infusion or subcutaneous LMWH at varying percentages of therapeutic doses (1.5 mg/kg for Enoxaparin and 200E/kg for Dalteparin) relative to bodyweight. For comparative analysis, the Mann–Whitney U test was applied to continuous data, while the Chi-square test was used for categorical data. All data analyses were conducted using the statistical software program R (version 4.1.2). Statistical significance was set at p < 0.05.

## Results

### Baseline data

**Fig. 1:**
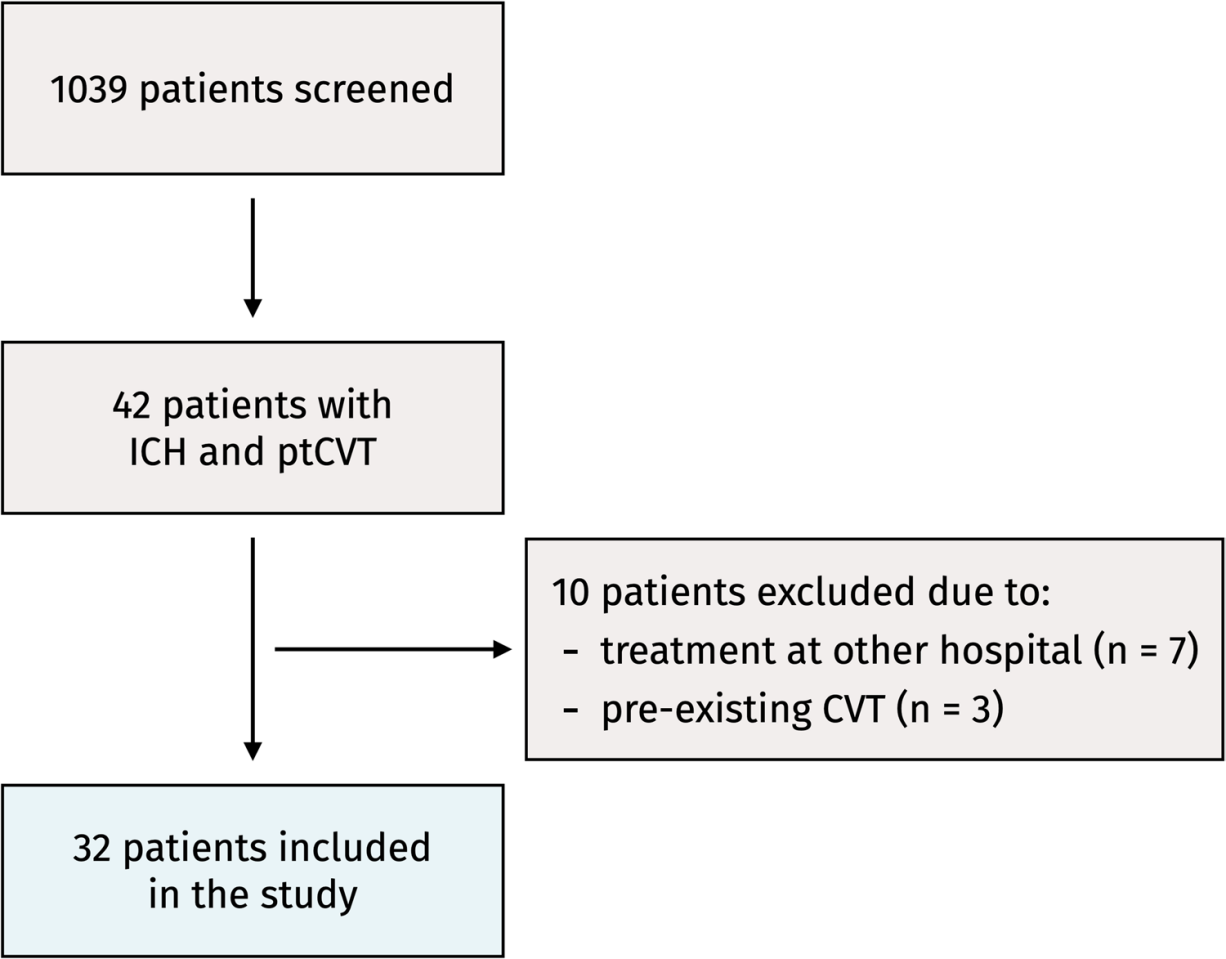
Selection of the study population

A total of 1,039 patients were screened, of whom 42 (4.0%) were diagnosed with a ptCVT. After exclusion due to incomplete records (*n* = 7) and pre-existing CVT (*n* = 3), 32 patients were included in the study (Fig. 1). Among the three cases of pre-existing CVT, two had known chronic CVT prior to the trauma, and one had a sigmoid sinus CVT that a hematologist determined had likely developed from an internal jugular vein totally implantable venous access device (Port-a-cath®) prior to the trauma.

The cohort was primarily male (69%), with a median age of 48 years (IQR 35 – 62) and weight of 82 kg (IQR 74 – 88). Severe TBI (GCS 3–8) constituted 53% of cases. No prior history of hypercoagulable states or anticoagulation was noted, although 3 patients were on antiplatelet treatment due to previous cardiovascular events. All patients had normal renal function on admission. The most common injury mechanisms were same-level falls (41%) and motor vehicle accidents (16%), and the median time from trauma to admission was 0.8 h (IQR 0.6 – 1.3) (Table 2).

**Table 2:** Baseline characteristics stratified by treatment strategy

Variable	All patients (*n* = 32)	LMWH < 50% (*n* = 16)	LMWH ≥ 50% (*n* = 8)	Heparin infusion (*n* = 8)	*p*-value
Age (years)	48 (35 – 62)	44 (39 – 63)	56 (30 – 63)	50 (23 – 60)	0.862
Female sex	10 (31%)	5 (31%)	3 (38%)	2 (25%)	> 0.999
Weight (kg)	82 (74 – 88)	83 (78 – 93)	84 (79 – 90)	70 (68 – 76)	0.032
Prior anticoagulant treatment	0 (0%)	0 (0%)	0 (0%)	0 (0%)	> 0.999
Prior antiplatelet treatment	3 (9.4%)	2 (12%)	1 (12%)	0 (0%)	0.794
GCS	8 (3 – 11)	10 (3 – 12)	6 (4 – 8)	8 (3 – 11)	0.792
Mild TBI	4 (12%)	2 (12%)	1 (12%)	1 (12%)	0.607
Moderate TBI	11 (34%)	7 (44%)	1 (12%)	3 (38%)	0.607
Severe TBI	17 (53%)	7 (44%)	6 (75%)	4 (50%)	0.607
Isolated TBI	23 (72%)	10 (62%)	7 (88%)	6 (75%)	0.535
Hours from injury to admission	0.8 (0.6 – 1.3)	0.8 (0.6 – 1.1)	0.7 (0.6 – 1.1)	1.0 (0.6 – 2.0)	0.838

Data is shown as median (interquartile range) or number (proportion). Abbreviations: *GCS* Glasgow Coma Scale; *LMWH* low-molecular-weight heparin; *ptCVT* post-traumatic cerebral venous thrombosis; *TBI* traumatic brain injury.

### Radiographic data

The median time from injury to ptCVT diagnosis was 24 h (IQR 10 – 39). All ptCVTs were caused by compression from either a fracture and/or extra-axial hematoma, with the exception of one iatrogenic case linked to a jugular bulb catheter. The majority of ptCVTs affected the sigmoid sinus (81%, *n* = 26) and/or the transverse sinus (66%, *n* = 21), with a small fraction involving the superior sagittal sinus (12%, *n* = 4). Bilateral dural ptCVTs were noted in just two instances, and in both cases at least one side had intact blood flow past the thrombus.

The most common coexisting traumatic ICH was an intraparenchymal hematoma (IPH) (*n* = 25), followed by subarachnoid hematoma (SAH) (*n* = 22), subdural hematoma (SDH) (*n* = 20) and epidural hematoma (EDH) (*n *= 5). The median hematoma volumes at ACT initiation were 9 ml (IQR 2 – 19) for IPH, 9 ml (IQR 6 – 28) for EDH and 8 ml (IQR 3 – 15) for SDH. Seven patients (22%) had showed hematoma expansion on the CT scan performed before ACT initiation. In addition, 21 (66%) patients underwent a craniotomy, with all but one performed prior to ACT initiation (Table 3).

**Table 3:** Radiographic data stratified by treatment strategy

Variable	All patients (*n* = 32)	LMWH < 50% (*n* = 16)	LMWH ≥ 50% (*n* = 8)	Heparin infusion (*n* = 8)	*p*-value
Hours from injury to ptCVT diagnosis	24 (10 – 39)	17 (7 – 30)	39 (19 – 60)	34 (16 – 58)	0.088
Bilateral ptCVT	2 (6.2%)	0 (0%)	1 (12%)	1 (12%)	0.433
ptCVT location					0.786
Superior sagittal sinus	4 (12%)	3 (19%)	1 (12%)	0 (0%)	-
Transverse sinus	21 (66%)	9 (56%)	6 (75%)	6 (75%)	-
Sigmoid sinus	26 (81%)	11 (69%)	8 (100%)	7 (88%)	-
ptCVT provocation	32 (100%)	16 (100%)	8 (100%)	8 (100%)	-
Jugular bulb catheter	1 (3.1%)	0 (0%)	1 (13%)	0 (0%)	-
Compression	31 (97%)	16 (100%)	7 (88%)	8 (100%)	-
Depressed skull fracture	5 (16%)	3 (19%)	0 (0%)	2 (25%)	-
Non-depressed skull fracture	13 (41%)	6 (38%)	4 (50%)	3 (38%)	-
Extraaxial hematoma	5 (16%)	3 (19%)	1 (12%)	1 (12%)	-
Combined hematoma and fracture	8 (25%)	4 (25%)	2 (25%)	2 (25%)	-
Traumatic ICH	32 (100%)	16 (100%)	8 (100%)	8 (100%)	-
IPH	25 (78%)	11 (69%)	7 (88%)	7 (88%)	0.627
IPH volume (ml)	9 (2 – 19)	15 (7 – 23)	6 (4 – 10)	1 (1 – 18)	0.207
EDH	5 (16%)	4 (25%)	0 (0%)	1 (12.5%)	0.399
EDH volume (ml)	9 (6 – 28)	8 (4 – 14)	-	49 (49 – 49)	0.400
SDH	20 (63%)	9 (56%)	6 (75%)	5 (63%)	0.889
SDH volume (ml)	8 (3 – 15)	9 (3 – 17)	12 (4 – 15)	4 (3 – 8)	0.630
SAH	22 (69%)	12 (75%)	6 (75%)	4 (50%)	0.491
Craniotomy	21 (66%)	10 (62%)	5 (62%)	6 (75%)	0.417
Hematoma expansion on CT performed before ACT initiation	7 (22%)	3 (19%)	3 (38%)	1 (12%)	0.313

Data is shown as median (interquartile range) or number (proportion). Abbreviations: *ACT* anticoagulation therapy; *EDH* epidural hematoma; *ICH* intracranial hematoma; *IPH* intraparenchymal hematoma; *LMWH* low-molecular-weight heparin; *ptCVT* post-traumatic cerebral venous thrombosis; *SAH* subarachnoid hematoma; *SDH* subdural hematoma.

### ptCVT treatment

All patients received ACT for their ptCVT. Treatment was initiated at a median of 41 h after trauma, corresponding to 9 h after ptCVT diagnosis. The median treatment dose for LMWH was 36% of the therapeutic dose (1.5 mg/kg for Enoxaparin or 200 E/kg for Dalteparin). Sixteen patients (50%) received LMWH at less than 50% of the therapeutic dose, ranging from 28 to 49%, while eight patients (25%) received LMWH at or above 50%, with doses between 50 and 72%. The remaining eight patients (25%) were treated with heparin infusions, with a median APTT range of 35–59 during the first 72 h.

Higher LMWH doses (≥ 50%) were predominantly administered in a split-dose regimen, with 88% of patients receiving this approach. In contrast, lower doses (< 50%) were more evenly divided, with 56% of patients receiving split doses and 44% receiving once-daily dosing.

No significant differences in baseline characteristics, radiographic findings, or outcomes were observed between the various treatment strategies (Tables 2, 3 and 4).

**Table 4:** Treatment and outcome data stratified by treatment strategy

Variable	All patients (*n* = 32)	LMWH < 50% (*n* = 16)	LMWH ≥ 50% (*n* = 8)	Heparin infusion (*n* = 8)	*p*-value
LMWH dose (% of treatment dose)	36 (31 – 54)	32 (28 – 36)	58 (54 – 64)	-	-
Split dosing	-	9 (56%)	7 (88%)	-	0.126
Hours from trauma to ACT initiation	41 (30 – 60)	32 (26 – 44)	45 (40 – 65)	56 (30 – 60)	0.124
APTT within 72 h of heparin infusion initiation	-	-	-	-	-
Lowest APTT	-	-	-	35 (34 – 37)	-
Highest APTT	-	-	-	59 (38 – 84)	-
Hematoma progression after ACT initiation	0 (0%)	0 (0%)	0 (0%)	0 (0%)	-
ptCVT complication					
Thrombosis progression	2 (6%)	2 (13%)	0 (0%)	0 (0%)	-
Venous infarct	1 (3%)	1 (6%)	0 (0%)	0 (0%)	-

Data is shown as median (interquartile range) or number (proportion). Abbreviations: *ACT* anticoagulation therapy; *APPT* activated partial thromboplastin time; *CT* computed tomography; *IPH* intraparenchymal hematoma; *LMWH* low-molecular-weight heparin; *SDH* subdural hematoma; *ptCVT* post traumatic cerebral venous thrombosis.

### Thrombotic complications

Three thrombotic complications occurred. The most serious was a venous infarct in a 38-year-old male with a transverse sinus thrombosis from a penetrating fracture. Despite identifying the thrombosis 20 h post-injury, Enoxaparin treatment at 36% of the therapeutic dose was delayed for an additional 17 h due to considerations for a potential facial nerve exploration. Even though a CT scan four days later showed a possible partial recanalization of the thrombosis, he suffered a partial venous infarction in the right cerebellum, but did not require posterior fossa decompression or hydrocephalus treatment.

Two cases of thrombosis progression were recorded. A 63-year-old male weighting 94 kg with a ptCVT in the right sigmoid and transverse sinus received 40 mg of Enoxaparin (28% of the therapeutic level) in a split-dose regimen. No change in the thrombus was seen on follow-up CT venography two days after starting ACT, but a repeat scan a week later revealed a small, new, non-occlusive CVT in the right jugular vein. This led to an increase in Enoxaparin to 56% of the therapeutic dose, which was well tolerated.

The second case of thrombus progression involved a 71-year-old male, who also weighed 94 kg, male with a CVT affecting the superior sagittal, transverse, and sigmoid sinuses. Diagnosed 30 h after injury, ACT was delayed by 17 h due to surgical evacuation of an ICH and SDH. Subsequent CT venography showed thrombosis enlargement. The patient then received Enoxaparin at 28% of the therapeutic dose for 20 days, later increased to the full therapeutic dose, which was well tolerated.

### Hemorrhagic complications

After assessing all follow-up CT scans, no cases of hematoma expansion following initiation of ACT were found.

## Discussion

This study evaluated thrombotic and hemorrhagic outcomes in TBI patients with concurrent ptCVT and ICH treated with ACT. All 32 patients received either LMWH or heparin infusions, making this the largest study to date involving patients with ptCVT and ICH treated with ACT. Among the cohort, thrombotic complications occurred in 3 patients, and notably, no patient experienced hematoma expansion after ACT initiation. These findings underscore the intricate balance required when managing the dual risks of thrombosis and hemorrhage, and suggests that ACT, when administered at reduced doses, may not increase the risk of hematoma expansion.

### Incidence of ptCVT

In this study, 4.0% of TBI patients were diagnosed with ptCVT. Each case was associated with predisposing factors such as compressing extra-axial hematomas, overlying fractures, or jugular bulb catheters. A previous study of severe TBI patients noted that all their ptCVT cases involved a skull fracture overlying the sinus [13], and a study on consecutive emergency department patients with TBI who underwent CT-venography revealed a ptCVT incidence of 41% among those with skull fractures extending to a dural sinus or jugular bulb [5]. While this underscores the role of skull fracture as risk factors for ptCVT development, is also suggest a diagnostic bias, as patients with these injuries are more likely to undergo CT venography, increasing the chances of ptCVT detection. Currently, the lack of routine CT venography in all TBI cases limits our understanding of the true incidence of ptCVT. However, routine venography for all TBI patients remains impractical due to resource constraints and radiation exposure, leaving us with an incomplete understanding of the true prevalence of ptCVT.

## Conservative vs active management

All patients in our study received ACT, which presents a contrast to Almandoz et al. [4], where none of the patients received anticoagulation. In their study, a thrombosis progression rate of 24% was observed among patients who underwent follow-up CT venography. In our cohort, thrombus progression occurred in only 6% of patients, with no bleeding progression observed. This suggests that ACT, when appropriately dosed, may offer advantages over conservative management when balancing the risks of thrombosis and hemorrhage. This perspective is further supported by the absence of bleeding progression in patients with ptCVT and ICH who were treated with LMWH or heparin in case reports by Grangeon et al. [12], Pescatori et al. [16], and Afshari et al. [1].

## ACT dosing strategy

Dosing decisions in our study were based on clinical judgment rather than strict adherence to a protocol, with LMWH doses ranging from 28 to 72% of the therapeutic level – lower than what is typically recommended for spontaneous CVT [7, 18]. This cautious approach could be more suitable for ptCVT, which is often due to mechanical compression rather than a hypercoagulable state. Of note, most patients with < 50% of the treatment dose only received thromboprophylaxis doses similar to what is typically given to most ICU-treated TBI patients within 48 h post-injury. For example, a patient weighing 70 kg treated with 5,000 units of Dalteparin or 40 mg of Enoxaparin would correspond to a 36% treatment dose. However, thrombus progression was observed in the patient who received the lowest dose of LMWH (28%), suggesting that this dosage might represent a lower threshold of effectiveness. This patient, who weighed 94 kg, was administered 40 mg of Enoxaparin, resulting in a relatively low effective treatment dose due to his large body weight. This case underscores the importance of individualized dosing of anticoagulants based on patient characteristics, such as body weight, to ensure effective DVT prophylaxis and prevent thrombus progression, as standard dosing may not always be sufficient.

In contrast, none of the patients treated with LMWH at doses above 50% experienced bleeding progression, indicating this level may effectively prevent thrombus growth without significantly increasing hematoma expansion risk. Furthermore, many patients were managed with a split-dose regimen, potentially reducing hemorrhagic complications by avoiding high peak plasma concentrations seen in once-daily dosing [3]. Split dosing also allows for more precise anticoagulation level adjustments, especially in cases of bleeding progression or when further surgeries may be needed.

## Timing of ACT initiation

The median time from injury to ptCVT diagnosis was 24 h, with ACT initiated at a median of 9 h post-diagnosis. This delay often ensured completion of any necessary craniotomy and stabilization of hematomas before treatment initiation. Patients given LMWH at doses under 50% started treatment sooner (median 31 h after injury) compared to those on higher doses (median 45 h), implying that conservative dosing enables earlier intervention. Heparin infusion, favored for its shorter half-life and the ability for swift cessation in the case of hematoma expansion, offers another feasible option in these circumstances. Notably, two out of the three thrombotic events in our cohort – the venous infarct and one instance of thrombus progression – occurred in patients whose ACT initiation was delayed by 17 h after ptCVT detection. This might indicate that a postponement in starting ACT could contribute to adverse thrombotic outcomes, highlighting the importance of timely treatment initiation.

### Role of heparin infusion

Heparin infusion was administered to eight patients in our cohort. Its rapid reversibility can be beneficial when the risk of hematoma progression is high. Our data suggest that heparin infusion was effective and safe, with no thrombotic or hemorrhagic complications observed. However, it requires frequent blood draws and dosage adjustments to make sure that APTT targets are met, which can be logistically challenging in a busy clinical setting. Additionally, interruption of the infusion can result in a rebound prothrombotic state, potentially exacerbating ptCVT. Therefore, we acknowledge that while heparin infusion can be used when rapid adjustment of anticoagulation is necessary, especially in the early post-trauma stages, LMWH at adjusted doses might offer an equally safe and effective alternative that may be preferred due to the logistical constraints of heparin infusions.

## Strengths and limitations

As a retrospective study, inherent biases such as selection and information biases are present. Without a control group of patients not receiving ACT, drawing definitive conclusions about treatment effectiveness is challenging. Moreover, in cases where ptCVT was diagnosed after prophylactic LMWH had been administered, the ACT initiation times reflect dose adjustments rather than the initial administration of ACT. The lack of access to complete coagulation laboratory values, including anti-Xa levels, also limited our ability to fully assess anticoagulant activity. The small sample size, though considerable in this field, also restricts the generalizability of the findings and precludes robust multivariate analyses to account for heterogeneity in GCS scores and hematoma types. Furthermore, distinguishing true thrombosis from luminal narrowing caused by external compression remains a challenge, though we believe this was less of an issue due to the independent review of imaging by two neuroradiologists during clinical care, along with a radiologist's re-evaluation for this study. Lastly, as a single-center study, the findings may not be broadly applicable to other institutions with different patient populations or treatment protocols. Prospective studies with larger sample sizes and a controlled design are warranted to and help establish more definitive clinical practice guidelines.

## Clinical implication of study results

Pending further validation, we propose three considerations for the management of patients with concurrent traumatic ICH and ptCVT. These are intended to strike a balance between anticoagulation and the risks of hematoma expansion, but require careful consideration of each patient's evolving clinical status.

1. Based on our findings, LMWH at approximately 50% of the therapeutic dose appears to be sufficient for ptCVTs caused by mechanical compression. Split-dose regimens may further reduce the risk of hemorrhagic complications.
2. Initiate LMWH as soon as hematomas are radiographically confirmed to be stable. In cases where hematomas remain unstable for several hours post-ptCVT diagnosis, starting with lower LMWH doses (between 30–50% of the therapeutic level) may offer a middle ground between the risk of hemorrhage and the benefit of anticoagulation, though this requires further validation.
3. Consider conducting routine CT/MRI venography to continuously assess the status of thrombosis and hematomas, allowing for timely adjustments to treatment as the patient’s condition evolves.

## Conclusion

This study highlights the delicate balance in managing concurrent traumatic ICH and ptCVT. Our findings suggest that a 50% therapeutic dose of LMWH effectively balances the reduction of clot progression with a minimized risk of hematoma expansion. Regular CT venography is essential for monitoring and guiding treatment adjustments. Further research is needed to refine these preliminary guidelines.

## Data Availability

Data will be made available by the corresponding author upon reasonable request.

## Abbreviations

ACT: Anticoagulation therapy
CT: Computed tomography
CVT: Cerebral venous sinus thrombosis
EDH: Epidural hematoma
IPH: Intraparenchymal hematoma
IQR: Interquartile range
GCS: Glasgow Coma Scale
LMWH: Low-molecular weight heparin
MRI: Magnetic resonance imaging
ptCVT: Post-traumatic cerebral venous sinus thrombosis
SAH: Subarachnoid hematoma
SDH: Subdural hematoma
TBI: Traumatic brain injury
